# Explore a novel function of human condensins in cellular senescence

**DOI:** 10.1186/s13578-020-00512-1

**Published:** 2020-12-29

**Authors:** Hongzhen Wang, Xin Liu, Guiying Li

**Affiliations:** 1 School of Life Sciences, Jilin Normal University, 136000 Siping, People’s Republic of China; 2 Key Laboratory for Molecular Enzymology and Engineering of the Ministry of Education, School of Life Sciences, Jilin University, 130012 Changchun, People’s Republic of China

**Keywords:** Human condensins, Cellular senescence, Oncogene-induced senescence, Replicative senescence

## Abstract

There are two kinds of condensins in human cells, known as condensin I and condensin II. The canonical roles of condensins are participated in chromosome dynamics, including chromosome condensation and segregation during cell division. Recently, a novel function of human condensins has been found with increasing evidences that they play important roles in cellular senescence. This paper reviewed the research progress of human condensins involved in different types of cellular senescence, mainly oncogene-induced senescence (OIS) and replicative senescence (RS). The future perspectives of human condensins involved in cellular senescence are also discussed.

## Introduction

Condensins were firstly identified for their fundamental roles in establishment and maintenance of mitotic chromosome condensation in cell-free system from Xenopus laevis eggs [1, 2]. Until now, most multicellular eukaryotes reported have two kinds of condensins, termed as condensin I and condensin II [3–6]. The two kinds of condensins are also exist in human cells [7–10]. Both human condensins are pentameric complexes composed of shared core SMC2/SMC4 (structural maintenance of chromosomes, SMC) heterodimer(also known as hCAP-E/hCAP-C)and three accessory non-SMC subunits, including a kleisin subunit and two HEAT-repeat proteins. They are hCAP-H(NCAPH), hCAP-D2(NCAP-D2) and hCAP-G(NCAPG) for condensin I and hCAP-H2(NCAP-H2), hCAP-D3(NCAPD3) and hCAP-G2(NCAP-G2) for condensin II [10–12].

The canonical roles of human condensins are participated in chromosome dynamics, including chromosome condensation and segregation during mitosis [7–12]. Although both human condensins have similar components and alphabetic structure, they show different nuclear distribution, localization on chromosomes and play distinct roles in chromosome dynamics during mitosis [9–13].

**Fig. 1:**
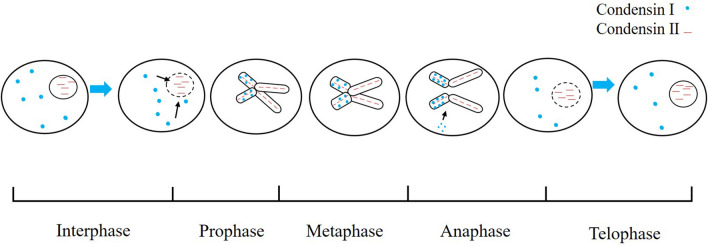
Different nuclear distribution, localization on chromosomes of human condensin I and condensin II during cell cycle (one chromosome is illustrated as an example)

In detail, during interphase condensin I is present in the cytoplasm, whereas condensin II is enriched within the nucleus [10, 14, 15]. During mitosis, initially condensin II participates in chromosome condensation within the nucleus in early prophase, whereas condensin I can interact with chromosomes only after the nuclear envelope breaks down [11]. Human condensin I shows a two-step dynamic binding. Once nuclear envelope breakdown, human condensin I rapidly associated with mitotic chromosomes then remained constant from prometaphase to late metaphase and chromatin bound human condensin I increased again just from anaphase onset until late anaphase when it dissociated from chromosomes[11, 12]. Similarly, human condensin I complexes dynamically bind to chromosomes in two steps during prometaphase and early anaphase whereas human condensin II complexes are stably bound to chromosomes throughout mitosis. Localization of human condensin II is centrally confined, but condensin I reaches ∼50% of the chromatid diameter from its center [16]. It is indicated that human condensin II but not condensin I is more indispensible for the salt-dependent, reversible reorganization of condensin II-based axes in chromosome shaping [17]. Moreover, human condensins show a discontinuous pattern along mitotic chromosomes and play a major role in controlling the elastic stiffness of metaphase chromosomes. Depletion of condensin II impacts chromosome mechanics more than depletion of condensin I and stiffness of the metaphase chromosome is more dependent on condensin II than on condensin I [18].This idea is somewhat inconsistent with a former study. It has been demonstrated that human condensin I but not condensin II can associate with KIF4A to confer rigidity to centromeres [19]. During anaphase, when human condensins are depleted, chromosomes are formed with improperly structured kinetochores and chromosome bridges appear in the cell [20]. Likewise, when human condensins are knocked down or dysfunction in human cells, chromatin bridges between daughter cells in anaphase and multiple nuclei in single cells are observed[21, 22]. During telophase, human condensins are involved in the mitotic chromosome conformation transformation into the interphase state as well. Recently, it is identified telophase as a critical transition between condensin- and cohesin-driven chromosome folding [23]. Consistently, human condensin II can initiate sister chromatid resolution during S phase [24]. Altogether, the differences in the timing of binding to chromosome and mutant phenotypes of dysfunction strongly indicated that human condensin I and II have fundamentally distinct functions during mitosis. Different nuclear distribution, localization on chromosomesof human condensin I and human condenisn II during cell cycle are shown as Fig. 1.

In addition to their mitotic functions, human condensins also play important roles in the prestressed condensed state of the nucleus, homologous recombination repair and gene expression during interphase [20, 24–35]. Beyond the multiple roles of human condensins mentioned above, increasing evidences show a novel function of human condensins in cellular senescence [36, 37].

Cellular senescence plays important protective roles in development, tissue homeostasis, wound healing, multiple age-related diseases and cancer [38, 39]. Cellular senescence is a stable state of irreversible cell cycle arrest caused by various forms of cellular stresses. Senescent cells lose DNA replication ability and still maintain cellular metabolic activity [40, 41]. Especially, cellular senescence can also be induced by pathophysiological stimuli, such as ROS (reactive oxygen species), oncogene activation, cytotoxic drugs and aging [38]. Nowadays four kinds of cellular senescence are recognized, i.e., oncogene-induced senescence (OIS), replicative senescence (RS), stress-induced premature senescence (SIPS) and therapy-induced senescence (TIS) [37, 38, 41, 42]. OIS is induced by oncogene expression and RS is induced by telomere shortening [37, 38, 41, 43–45]. SIPS is induced by various external signals, such as UV, hyperoxia, hydrogen peroxide, etc. [41, 45]. TIS is caused by traditional cancer therapy and it can be an effective way to treat cancer while lessening side effects [42, 46–48].

Although human condensins play classical roles in chromosome dynamics during mitosis, their nonmitotic functions have been payed more and more attention than before. As a novel function of human condensins in cellular senescence during interphase, much more problems remain to be further explored. This paper reviewed the research progress of human condensins involved in different types of cellular senescence, mainly oncogene-induced senescence (OIS) and replicative senescence (RS). The future perspectives of human condensins in cellular senescence are also discussed.

### Human condensins involved in cellular senescence

Firstly, human condensins are involved in OIS. Initially, human condensin II is found to play a novel role in OIS. Overexpression of human condensin II, but not human condensin I, induces cellular senescence and senescence-associated heterochromatic foci (SAHF) formation and depletion of human condensin II inhibits the establishment of OIS [36]. In detail, the N-terminus truncated variant, hCAP-H2ΔN (lacking the first 50 amino acids) is mostly localized at the nuclear matrix and accumulates in quiescent and senescent cells. The ΔN variant exists as an insoluble nuclear structure while the full-length hCAP-H2 associates with mitotic chromosome. Overexpression of the full-length hCAP-H2 and ΔN variant can significantly induce senescence. Expression of hCAP-H2ΔN was increased during OIS. Moreover, hCAP-H2 knockdown (KD) also inhibited Ras-induced senescence. It is suggested that human condensin II drives senescence via nuclear/genomic reorganization [36]. Recently, the roles of human condensin II are reported to participate in cellular senescence through compartmental reorganizations coupled to gene regulation [37]. Human condensins strengthen and expand euchromatic A compartments and promote/maintain BA transitions upon senescence. Concretely, localization of hCAP-H2 is firstly studied and the results show they localize at active senescence genes, highly transcribed housekeeping genes, and potential enhancers. Next, by compared the general organization of the human genome into A and B compartments in OIS and growing cells, it is found that A compartments in OIS cells were significantly enlarged (~ 50%) compared to counterparts in growing cells. The increased BA transitions result in the significant enlargement of A compartments in OIS cells compared to growing cells. In efforts to find a functional link between senescence-dependent compartmental reorganizations and condensin, the following results show that human condensin II binding and dissociation are involved in BA and AB transitions and this mechanism is conserved between OIS and RS. In addition to promoting and maintaining BA transitions, human condensin II also play important roles to maintain euchromatic A compartments and facilitate genomic contacts in A compartments, that is, to reinforce A compartments. Further research suggests that human condensin II plays a direct role in the upregulation of senescence genes because that many genes upregulated upon OIS and downregulated by hCAP-H2 KD (knock down) are hCAP-H2 binding genes. Collectively, human condensins are not only involved in euchromatic A compartments and BA transitions, but also involved in the upregulation of senescence genes upon senescence. Therefore, the roles of human condensin II in cellular senescence may be through compartmental reorganizations coupled to gene regulation [37].

Secondly, human condensins are involved in RS. SMC2 and SMC4, core subunits of human condensins, are demonstrated to be down-regulated in the serially passaged fibroblast cells by proteomic study and they are supposed to play an important role in RS [41]. Consistently, several subunits of human condensin I and II (mainly non-SMC subunits, i.e., hCAP-D2, hCAP-D3, hCAP-G, hCAP-G2, hCAP-H and SMC4) are downregulated in *KDM3A*- or *KDM4C*-knockdown human umbilical cord-derived stromal cells (hUCMSCs) or upregulated in *KDM3A* or *KDM4C*-overexpressing hUCMSCs (49). Especially, *hCAPD-2* and *hCAPG-2* are positively regulated by KDM3A and KDM4C with their H3K9 demethylase activity and human condensins regulated by *KDM3A* or *KDM4* might be critical for stability of the heterochromatin structure during senescence. Recently,as mentioned above, human condensin II is involved in BA and AB transitions not only during OIS but also during RS [37].

Hitherto, there are few literatures published from the point that human condensins are involved in stress-induced premature senescence (SIPS) and therapy-induced senescence (TIS) [36, 37, 49]. Abnormal expression of subunits of human condensins and changes of structure of chromosomes in four types of cellular senescence are shown in Table 1.

**Table 1:** Abnormal expression of subunits of human condensins and changes of structure of chromosomes in four types of cellular senescence

Types of cellular senescence	Abnormal expression of subunits of human condensins	Changes of structure of chromosomes	Number of literature
OIS	hCAPH2↑ hCAPH2ΔN↑ SMC2↓, SMC4↓	Formation of senescence-associated heterochromatic foci (SAHF), drives senescence via nuclear/genomic reorganization; strengthen and expand euchromatic A compartments and promote/maintain BA transitions upon senescence	[36, 37, 41 (Supplementary Fig. 8)]
RS	SMC2↓ SMC4↓ hCAP-D2(NCAPD2)↓ hCAP-D3(NCAPD3)↓ hCAP-G(NCAPG)↓ hCAP-G2(NCAPG2)↓ hCAP-H()(NCAPH)↑, hCAPH2((NCAPH2)↑	SAHF were not detected in RS cells, the sizes of both A and B compartments became significantly enlarged and the numbers of A and B compartments decreased; heterochromatin reorganization to restrain DNA damage and progression of MSC senescence via transcriptionally activating human condensins; telomere shortening	[37, 41, 43–45, 49 (Supplementary Figure S4)]
SIPS	Unpublished		[37, 41, 49]
TIS	Unpublished		[37, 41, 49]

“↑” is a symbol as upregulation of expression, “↓” is a symbol as downregulation of expression

## Conclusions

Apart from the multiple canonical functions of chromosome dynamics played by human condensins during mitosis and interphase, increasing evidences show a novel function of human condensins in cellular senescence. Human condensins play important roles in the main two types of cellular senescence, i.e. oncogene-induced senescence (OIS) and replicative senescence (RS).

### Future perspectives of human condensins in cellular senescence

To explore a novel function of human condensins in cellular senescence, three future perspectives are presented as follows.

Firstly, how human condensins involved in interphase nuclear reorganization during cellular senescence needs further study. Both human condensin I and II are required for maintenance of the interphase nuclear architecture. Human condensins regulated by KDM3A or KDM4 might be critical for stability of the heterochromatin structure during senescence [49]. Depletion of human condensin II leads to dramatic disruption of nuclear architecture and nuclear size [50]. Interaction between human condensin II and SAHF provide an additional platform for studies on condensins participating dynamic interphase chromatin reorganization [51]. Recently, human condensin II subunit hCAP-H2 is demonstrated to associate with shelterin protein TRF1 and be required for telomere stability [52]. Furthermore, there is small fraction of human condensin I retained and be active in both gene regulation and chromosome condensation in interphase nuclei [53–55]. Although human condensin II is reported to be involved in SAHF formation and BA and AB transitions, whether human condensin I involved these process is still unclear [36, 37]. It is also intriguing to explore what roles of human condensin I play in cellular senescence during interphase.

Secondly, the different functions and possible interplays of human condensins in cellular senescence, quiescence and carcinogenesis needs further study. Although cell cycle differences exist between cellular senescence and quiescence, either cellular senescence or quiescence misregulation is implicated in cancer progression [39, 42, 56, 57]. It is surprising that condensins are involved in all the three processes [36, 37, 41, 49, 58–62]. In a mutant mouse carrying a constitutive missense mutation in the condensin II kleisin-β subunit *Caph2*, the mutation specifically causes chromatin decondensation and condensin II is demonstrated to be required for peripheral T-cell development and maintenance of the quiescent state [58–60]. With further analysis, the mutant mice show condensin II-dependent thymic lymphomas formation through tissue-specific genome instability [61]. Recently, in the quiescent state of Saccharomyces cerevisiae, condensin is required for widespread transcriptional silencing and dramatic chromatin condensation through binding throughout the budding yeast genome and induces the formation of large chromatin loop domains [62]. Hitherto, apart from cellular senescence and tumorgenensis, whether human condensins play a role in quiescence is unclear. In addition, how human condensins involved the three processes need be extensively explored [41, 49, 52, 62]. It is promising to explore possible interplays of cellular senescence and tumor formation and anticancer therapy from the viewpoints of human condensins [63–65].

Finally, whether some crosstalks exist in condensins, cohesins and SMC5/SMC6 complexes is yet unclear during human cellular senescence. SMC complexes have ancient origins and share structural similarities. Condensin, cohesin and SMC5/SMC6 complex are three types of evolutionarily conserved SMC complexes within eukaryotic cells and the three complexes are all reported to be involved in cellular senescence in different organisms. Concretely, loss of the recruitment cohesin and condensin I complexes to pericentromeric regions causes to block efficient repair of the regions and leads to formation of persistent DNA damage foci in senescent human adult stem cells [66]. Depletion of human SMC5/6 subunits by RNAi inhibits telomere homologous recombination and causing telomere shortening and cellular senescence in human ALT (ALT, alternative lengthening of telomeres) cells [67, 68]. Similarly, SMC5/SMC6 complex as a target of Mms21-dependent sumoylation is also involved in cellular senescence in *Saccharomyces cerevisiae* [69]. Of note, interaction of condensin and cohesin is reported as a chromosome folding intermediate during telophase as a critical transition between condensin- and cohesin-driven chromosome folding [23]. Distinct roles of cohesin and condensin are required in the establishment of 3D nuclear organization in Drosophila [70]. Common kleisin-hinge interaction and different modes of regulation are also proposed in condensin and cohesin in fission yeast [71]. Likewise, functional interplay between cohesin and Smc5/6 complexes is reviewed for significant overlap of their location, function and crosstalk between these two complexes [72]. Similarly, there are also papers about interactions with condensin and Smc5/6 complexes. Depletion of Smc5 and Smc6 results in abnormal distribution of condensins and chromosome segregation errors in human cells [73]. Furthermore, mutation of SMC5 also leads to abnormal distribution of condensin along chromosomes with decreased condensin accumulation at pericentromeric regions and enrichment of condensin on chromosome arms in mouse embryonic stem cells (mESCs) [74]. There are complex chromatin reorganizations in senescent cells [75–77]. Recently, different models of SMC complex function have been presented [78–80]. Based on all mentioned above, we propose that senescence-associated chromatin reorganization may need cooperative function of the three SMC complexes, but the molecular mechanism is yet unknown.

Taken together, human condensins play important roles in cellular senescence. It is worthy to be explored for the new multifunctions of human condensins in cellular senescence.

## Data Availability

Not applicable.
